# Substructurability: the effect of interface location on a real-time dynamic substructuring test

**DOI:** 10.1098/rspa.2016.0433

**Published:** 2016-08

**Authors:** N. Terkovics, S. A. Neild, M. Lowenberg, R. Szalai, B. Krauskopf

**Affiliations:** 1 Faculty of Engineering, University of Bristol, Bristol BS8 1TR, UK; 2 Department of Mathematics, University of Auckland, Auckland 1142, New Zealand

**Keywords:** substructurability, delay differential equation, phase margin

## Abstract

A full-scale experimental test for large and complex structures is not always achievable. This can be due to many reasons, the most prominent one being the size limitations of the test. Real-time dynamic substructuring is a hybrid testing method where part of the system is modelled numerically and the rest of the system is kept as the physical test specimen. The numerical–physical parts are connected via actuators and sensors and the interface is controlled by advanced algorithms to ensure that the tested structure replicates the emulated system with sufficient accuracy. The main challenge in such a test is to overcome the dynamic effects of the actuator and associated controller, that inevitably introduce delay into the substructured system which, in turn, can destabilize the experiment. To date, most research concentrates on developing control strategies for stable recreation of the full system when the interface location is given *a priori*. Therefore, substructurability is mostly studied in terms of control. Here, we consider the interface location as a parameter and study its effect on the stability of the system in the presence of delay due to actuator dynamics and define substructurability as the system’s tolerance to delay in terms of the different interface locations. It is shown that the interface location has a major effect on the tolerable delays in an experiment and, therefore, careful selection of it is necessary.

## 1. Introduction

For many engineering structures, full-scale physical tests are not achievable; for example, if the size of the structure is large or the related costs make the test impractical, or simply because not all of the components are available for testing at the appropriate point in the design process. Although one could test individual substructures of the full system, it cannot be guaranteed that the substructure always behaves in exactly the same way when it is part of an overall structure, neither can the behaviour of the overall structure be easily deduced if there is nonlinear coupling between the substructures.

**Figure 1.**
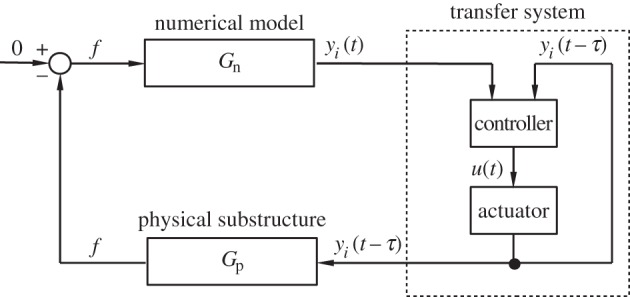
Schematic of a real-time dynamics substructuring test. The numerically calculated displacement at the interface *y*_*i*_(*t*) feeds into the physical substructure via the actuator, which introduces the delay *τ* to the system. The delayed input to the physical substructure *y*_*i*_(*t*−*τ*) results in a delayed force feedback to the numerical model.

Real-time dynamic substructuring has been developed to help overcome these challenges. In a substructuring test, part of the physical system is tested experimentally and the remainder is modelled numerically. The physical part is usually the one that, for some reason, is difficult to model mathematically. The part of the system that can be modelled with greater confidence, on the other hand, is represented numerically in the experiment. Advanced real-time control techniques are then used to effectively ‘glue together’ the experimental test specimen and the numerical model of the remainder of the system at their interface, via a *transfer system*. A simple transfer system may consist of a force sensor, an actuator and their controller. The sensor is responsible for sending the force feedback to the numerical model, and the actuator imposes the interface displacement, which is computed by the numerical model, to the physically tested component (figure 1). Through displacement control of the actuators and the force feedback to the numerical model, the physical–numerical interface can be matched, so that the dynamics of the overall system is replicated [1,2].

The method was developed from the so-called pseudo-dynamic testing method. This is already a hybrid method, whereby a computer is connected online to the test specimen, and it is used to calculate displacements based on a numerically prescribed excitation profile and the directly measured restoring forces of the structure. The displacements are then fed back to the specimen quasi-statically [3–7]. The main drawbacks of the pseudodynamic test method are that any rate-dependent characteristics of the test structure are only considered numerically. For applications where rate-dependent behaviour—i.e. damping or inertia—is important, a real-time test is necessary and, therefore, a quasi-static test is insufficient. This requirement led to the development of the real-time dynamic substructuring test method.

As opposed to the pseudodynamic test, in a real-time dynamic substructuring test, the numerical calculations are performed within the time interval between the load increments. With accurate control of the output, this ensures that the rate-dependence is captured correctly during the test. However, this new requirement means very short processing times and, therefore, calls for fast and efficient integration algorithms as well as very accurate control. The first publication on performing a successful real-time dynamic substructuring test is by Nakashima *et al*. [8]. Further developments of the method include applications to multi-actuator systems [9–11] and the assessment of the control challenges associated with the technique [12,13]. A schematic of a real-time dynamic substructuring test can be seen in figure 1.

The main challenge of a real-time dynamic substructuring test is to ensure that the displacements and forces at either side of the interface between the numerical and physical substructures match. However, unless the controller is perfect, the transfer system will introduce errors into the substructuring scheme because the displacement imposed on the physical substructure will not be exactly that demanded by the numerical model.

In this work, we model the overall transfer system error as a constant, state-independent delay. Hence, the substructured system may be modelled and studied as a functional differential equation [14]. This approach is widely used and proved to be an efficient way of showing how the delay affects the overall feedback system (e.g. [15,16]). Its validity is based on the consideration of the main three sources that typically contribute to the transfer system error in substructuring.

Firstly, due to the inertial effects of the actuator there is an inevitable amplitude and phase error between the input and output of the actuator. The transfer system controller can easily overcome the amplitude errors introduced by the actuator dynamics, but generally a residual phase error or lag remains [12]. It is common for this phase lag to be modelled as a constant delay; this is considered accurate because, typically, the excitation frequency band in a substructuring test is narrow and so the frequency dependency of the actuator dynamics is negligible. Examples of where this approximation has been used include [1,2], and its validity is further supported by successful applications of delay compensation techniques to reduce it (e.g. [15,17]).

Secondly, the sampling procedure of the digital controller introduces a delay into the scheme that, in general, is not constant. However, if the sampling frequency is sufficiently high when compared with the time scale of the actuator dynamics (as is the case in the set-up considered here), it may be approximated as constant [18].

Thirdly, the computational time of the numerical model results in a delay of the calculated displacement relative to the force signal that is being fed back from the physical substructure. While the required computational time may depend on the state of the system, this delay is generally considered to be constant. Indeed, this can be achieved in practice by enforcing a constant (maximal) computation time.

Taken together, these three main causes of transfer system error can be modelled as a single delay that is state-independent and constant in very good approximation. Finally, we mention that another challenge in actuator control is real-time noise and its effect on the robustness of the control (e.g. [19]); however, we assume here that this stochastic effect is sufficiently small, and it is not included in this study.

The term *substructurability* was first mentioned in 2005 by Neild *et al.* [12], who experimentally study a substructured mass–spring–damper system, with different portions of the mass being the physical substructure. In a similar manner to controllability and observability, substructurability is defined as the ability to achieve a stable substructuring configuration as indicated by the system’s response to interface delay. They observed that the choice of the ‘interface location’ seemed to affect the overall performance of the substructuring test. This is the definition we use here. To avoid confusion, it is important to note that the term *substructurability* has been defined differently in a similar control-related context, where again the aim is to give a measure of the suitability of the overall substructuring scheme: Tu & Jiang [19] define substructurability in terms of selecting suitable control parameters, for a given numerical–physical interconnection, and the effect of ill-conditioning on the scheme.

Following the substructurability definition by Neild *et al*. [12], the aim here is to understand and assess what effect the interface location has on the delay threshold of the substructured system. To this end, we employ both analytical methods and then more generally applicable transfer function analysis. The paper is structured as follows. In §§2 and 3, a substructured two degree-of-freedom system is studied in terms of stability, considering two different interface locations. These systems are studied both analytically and then by transfer function analysis. In §4, the effect of interface location is studied on a continuously distributed mass system, namely a clamped cable. This allows us to show the effect of the interface location on the stability of a substructured system more generally.

## 2. Substructurability of a two degree-of-freedom system

**Figure 2.**
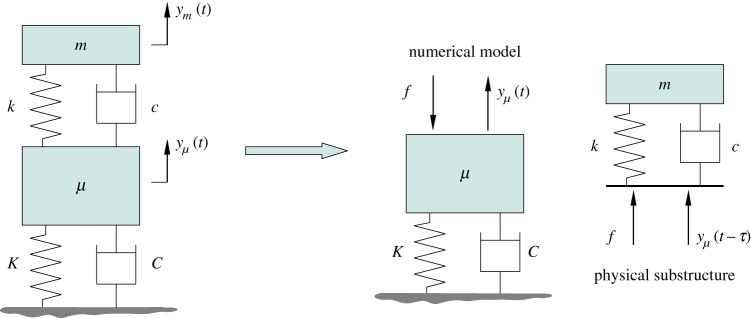
Schematic of the substructured two degree-of-freedom model. The numerical part consists of the mass–spring–damper unit fixed to the ground and the physical substructure is the mass–spring–damper unit on the top of the structure. (Online version in colour.)

First, we consider a conceptually simple substructuring set-up with a two degree-of-freedom mass–spring–damper. This model is used to demonstrate the methods for the analysis, and it reveals how a simple substructured system is affected by the interface location. To begin with, the system is separated by the interface into two structurally identical units (figure 2). The physical substructure is the upper mass–spring–damper unit. The two masses are *μ* and *m*, the stiffness of the springs are *K* and *k* and the damping coefficients are *C* and *c*. The calculated displacement from the numerical model is *y*_*μ*_(*t*) and the displacement imposed on the physical substructure is some past value of this calculated displacement, *y*_*μ*_(*t*−*τ*), where *τ* is the constant time delay. The feedback signal to the numerical model is the force, *f*, required to impose the displacement on the physical substructure. The equations of motion of the system being emulated read

2.1 $$\begin{array}{rl} & {y_{\mu }}^{\prime \prime }+2\zeta_{\mu }{y_{\mu }}^{\prime }+y_{\mu }-2\zeta_{m}({y_{m}}^{\prime }-{y_{\mu }}^{\prime })-\kappa (y_{m}-y_{\mu })=0\\ \;\text{and}\; & p{y_{m}}^{\prime \prime }+2\zeta_{m}({y_{m}}^{\prime }-{y_{\mu }}^{\prime })+\kappa (y_{m}-y_{\mu })=0. \end{array}\}$$

Here, the *prime* refers to differentiation with respect to the non-dimensional time variable T=*ω*_*n*_*t*, where $\omega_{n}=\sqrt{K/\mu }$. Furthermore, the non-dimensional parameters are given by

$$\kappa =\frac{k}{K},\;p=\frac{m}{\mu },\;\zeta_{\mu }=\frac{C}{2\sqrt{K\mu }}\;\mathrm{and}\;\zeta_{m}=\frac{c}{2\sqrt{K\mu }}.$$

In this substructuring configuration, the interface displacement of the physical substructure is delayed by *τ* relative to the interface displacement of the numerical part to capture the effects of delay due to the actuator and associated controller (figure 2). In the non-dimensional equations of motion this means that, in the terms corresponding to the physical part, the displacement *y*_*μ*_ and its derivatives are delayed with the non-dimensional delay T, where $T=\omega_{n}\tau$. The equations of motion of the substructured system are, therefore, given by

$$\begin{array}{rl} & y_{\mu }^{\prime \prime }(T)+2\zeta_{\mu }y_{\mu }^{\prime }(T)+y_{\mu }(T)-2\zeta_{m}(y_{m}^{\prime }(T)-y_{\mu }^{\prime }(T-T))-\kappa (y_{m}(T)-y_{\mu }(T-T))=0\\ \;\text{and}\; & py_{m}^{\prime \prime }(T)+2\zeta_{m}(y_{m}^{\prime }(T)-y_{\mu }^{\prime }(T-T))+\kappa (y_{m}(T)-y_{\mu }(T-T))=0. \end{array}\}$$

which, in vector form, reads

2.2 $${\text{x}}^{\prime }(T)=\text{A}\text{x}(T)+\text{B}\text{x}(T-T).$$

Here,

$$\text{A}=\left[\begin{array}{cccc} 0 & 0 & 0 & 0\\ -1 & -2\zeta_{\mu } & \kappa & 2\zeta_{m}\\ 0 & 0 & 0 & 1\\ 0 & 0 & -\frac{\kappa }{p} & -2\frac{\zeta_{m}}{p} \end{array}\right]\;\mathrm{and}\;\text{B}=\left[\begin{array}{cccc} 0 & 0 & 0 & 0\\ -\kappa & -2\zeta_{m} & 0 & 0\\ 0 & 0 & 0 & 0\\ \frac{\kappa }{p} & 2\frac{\zeta_{m}}{p} & 0 & 0 \end{array}\right],$$

and the state vector is

2.3 $$\text{x}=[y_{\mu }(T),\;y_{\mu }^{\prime }(T),\;y_{m}(T),\;y_{m}^{\prime }{\left(T)\right]}^{T}.$$

Equation (2.2) is a functional differential equation of retarded type or as it is more often referred to: a delay differential equation. Let us study how the delay affects the stability of the originally stable emulated system. Before considering a more generally applicable method later in this section, first we analyse the system analytically by determining the roots of the characteristic equation and their stability.

### (a) Analytical time-domain analysis

The characteristic equation is derived by substituting a trial solution into (2.2) in the form of $\text{x}=\text{K}e^{\lambda T},\text{K}\in C^{4},\text{x}\in C$. The substitution leads to the equation

$$(-\lambda \text{I}+\text{A}+\text{B}e^{-\lambda T})\text{K}e^{\lambda T}=\text{0},$$

from which, the characteristic equation is given by

2.4 $$\begin{array}{rl} C(\lambda ;\varrho ) & =\lambda^{4}+2\left(\zeta_{\mu }+\frac{\zeta_{m}}{p}\right)\lambda^{3}+\left(1+\frac{\kappa }{p}+\frac{4\zeta_{\mu }\zeta_{m}}{p}\right)\lambda^{2}+2\left(\frac{\kappa }{p}\zeta_{\mu }+\frac{\zeta_{m}}{p}\right)\lambda \\ & \;+\frac{\kappa }{p}+(2\zeta_{m}\lambda^{3}+\kappa \lambda^{2})e^{-\lambda T}=0. \end{array}$$

Here *ϱ*=[*κ* *p* *ζ*_*μ*_ *ζ*_*m*_] is the array of the non-dimensional parameters.

Owing to the transcendental nature of (2.4), there are infinitely many roots. For the corresponding system to be asymptotically stable, all of these roots need to have negative real parts. At the limit of stability, some of the characteristic roots have zero real parts, for some critical values of the parameters. As λ=0 is not a solution of (2.4), the only way for the roots to have zero real part is when a complex conjugate pair of roots is located on the imaginary axis, that is, if λ_1,2_=±i*Ω*, $\Omega \in R^{+}$. This means that a system with this linearization experiences Hopf bifurcations when higher-order terms are present.

#### (i) Stability boundaries

Upon substituting λ_1_=i*Ω* into (2.4) for the critical set of parameters *ϱ*_c_, separating the real and imaginary parts of the function and eliminating T from the resulting trigonometrical equations, the final equation for the critical eigenvalues becomes

2.5 $$\begin{array}{rl} & \Omega^{8}-2\left(1+\frac{\kappa }{p}+2(\zeta_{m}^{2}-\zeta_{\mu }^{2})\right)\Omega^{6}+\left(1+\frac{\kappa^{2}}{p^{2}}-\kappa^{2}+4\frac{\kappa }{p}(1-2\zeta_{\mu }^{2}\right)\\ & \;-8\frac{\zeta_{\mu }\zeta_{m}}{p^{2}}(1-2\zeta_{\mu }\zeta_{m}))\Omega^{4}-2\left(\frac{\kappa }{p}+\frac{\kappa^{2}}{p^{2}}(1-2\zeta_{\mu }^{2})-4\frac{\zeta_{m}^{2}}{p^{2}}\right)\Omega^{2}+\frac{\kappa^{2}}{p^{2}}=0. \end{array}$$

The above equation is a fourth-order polynomial in *Ω*^2^. Furthermore, the expression for the non-dimensional delay T associated with the critical eigenvalues is given by

2.6 $$T=\frac{1}{\Omega }\left[\tan^{-1}\left(\frac{-\Omega^{4}+(1+\kappa /p+4\zeta_{\mu }\zeta_{m}/p)\Omega^{2}-\kappa /p}{2(\zeta_{\mu }+\zeta_{m}/p)\Omega^{3}-2((\kappa /p)\zeta_{\mu }+\zeta_{m}/p)\Omega }\right)+2n\pi -\tan^{-1}\left(\frac{\kappa }{2\zeta_{m}\Omega }\right)\right],$$

where *n* is a non-negative integer. Because $n\in \{0\}\cup Z^{+}$, for each non-negative root of (2.5) there exist an infinite number of T for which the respective eigenvalue is at the limit of stability.

Owing to the high order of the polynomial, the roots of (2.5) can only be studied in a convenient manner algebraically in the undamped case, that is, when *ζ*_*m*_=*ζ*_*μ*_=0. In this case, the roots are given by

2.7 $$\begin{array}{rl} \Omega_{1,2}^{2} & =\frac{1}{2}\left(\kappa \left(\frac{1}{p}-1\right)+1\right)\pm \frac{1}{2}\sqrt{{\left(\kappa -1\right)}^{2}-\frac{2\kappa }{p}(\kappa +1)+\frac{\kappa^{2}}{p^{2}}}\\ \;\text{and}\;\Omega_{3,4}^{2} & =\frac{1}{2}\left(\kappa \left(\frac{1}{p}+1\right)+1\right)\pm \frac{1}{2}\sqrt{{\left(\kappa +1\right)}^{2}+\frac{2\kappa }{p}(\kappa -1)+\frac{\kappa^{2}}{p^{2}}}. \end{array}\}$$

Note that if κ→∞ the system being emulated becomes structurally identical to that studied in [16], where a single mass–spring–damper was considered with a portion of the mass taken as the physical substructure. In that work, the authors conclude that the mass ratio of the numerical and physical parts play an important role in terms of the stability of the substructured system. Therefore, in this work too, the stability and, hence, the solutions for (2.7), are considered in terms of the mass ratio *p*.

In order for *Ω*^2^ to be real, the discriminants in (2.7) must be non-negative for all positive values of *κ*. For $\Omega_{1,2}^{2}$, the condition ensuring this is given by

2.8 $$p\leq \frac{1+\kappa -2\sqrt{\kappa }}{{\left(\kappa -1\right)}^{2}}\kappa \;\text{or}\;\frac{1+\kappa +2\sqrt{\kappa }}{{\left(\kappa -1\right)}^{2}}\kappa \leq p.$$

For $\Omega_{3,4}^{2}$, the discriminant is non-negative for all non-negative values of *p*. Furthermore, assuming the criteria in (2.8), the roots $\Omega_{1,2}^{2}$ are always positive, since the condition given by

$$\kappa \left(\frac{1}{p}-1\right)+1>\sqrt{{\left(\kappa -1\right)}^{2}-\frac{2\kappa }{p}(\kappa +1)+\frac{\kappa^{2}}{p^{2}}}$$

always holds.

Further, for fixed values of *κ* there may be two, three (one of which is repeated) or four positive values of *Ω* depending on the value of *p*. Moreover, for the case of *ζ*_*m*_=0, the expression in (2.6) becomes singular. Therefore, the limit of T as *ζ*_*m*_ approaches zero (while *ζ*_*μ*_=0) needs to be considered. This, for the undamped case in general, is given by

$$\lim_{\zeta_{m}\to 0}T=\frac{2n\pi }{\Omega }.$$

In particular, for *Ω*_1−4_, the corresponding values of $T_{1-4}^{n}$ are given by

2.9 $$T_{1,2}^{n}=\frac{(2n+1)\pi }{\Omega_{1,2}},\;T_{3}^{n}=\frac{2n\pi }{\Omega_{3}}\;\text{and}\;T_{4}^{n}=\frac{(2n+2)\pi }{\Omega_{4}},\;n\in \{0\}\cup Z^{+}.$$

#### (ii) Stability analysis

After deriving the expression for the critical time delays, the stability of the undamped system needs to be analysed as T varies. To this end, the eigenvalues of (2.4) are considered as functions of T and the sign of the derivative of the real part of them is determined at the points where λ(T) is pure imaginary. If the derivative is positive, it means that, as T increases, λ crosses the imaginary axis from the left to the right, which means that the equilibrium loses stability. On the other hand, if the derivative is negative, the stability of the equilibrium is regained. This technique is widely used to determine stability before and after passing Hopf bifurcation points (e.g. [20,16]).

If λ=λ(T), the derivative of the characteristic function (2.4) with respect to T is given as

2.10 $$\frac{dC}{dT}=\left(4\lambda^{3}+2\left(1+\frac{\kappa }{p}\right)\lambda +\kappa \lambda (2-\lambda T)\;e^{-\lambda T}\right)\frac{d\lambda }{dT}-\kappa \lambda^{3}\;e^{-\lambda T}=0.$$

From (2.10), the expression for ${\left(d\lambda /dT\right)}^{-1}$ may be written as

2.11 $${\left(\frac{d\lambda }{dT}\right)}^{-1}=\frac{\left(4\lambda^{3}+2(1+\kappa /p)\lambda +\kappa \lambda (2-\lambda T)\;e^{-\lambda T}\right)}{\kappa \lambda^{3}\;e^{-\lambda T}}.$$

Moreover, $e^{-\lambda T}$ can be re-expressed using (2.4) as

$$e^{-\lambda T}=\frac{\lambda^{4}+(1+\kappa /p)\lambda^{2}+\kappa /p}{\kappa \lambda^{2}}.$$

With this expression, equation (2.11) can be written as

2.12 $${\left(\frac{d\lambda }{dT}\right)}^{-1}=-\frac{T\lambda^{5}+2\lambda^{4}+T(1+\kappa /p)\lambda^{3}-\kappa (2-T\lambda )}{(\lambda^{4}+(1+\kappa /p)\lambda^{2}+\kappa /p)\lambda^{2}}.$$

Using the identity

$${\mathrm{sgn}\left(\frac{d\;ℜ(\lambda )}{dT}\right)|}_{\lambda =i\Omega }={\mathrm{sgn}\left(ℜ{\left(\frac{d\lambda }{dT}\right)}^{-1}\right)|}_{\lambda =i\Omega }$$

and upon substituting λ=i*Ω* into (2.12), the expression reads

2.13 $${\mathrm{sgn}\left(\frac{d\;ℜ(\lambda )}{dT}\right)|}_{\lambda =i\Omega }=\mathrm{sgn}\left(-\frac{2(\kappa /p-\Omega^{4})}{\Omega^{2}(\Omega^{4}-(1+\kappa /p)\Omega^{2}+\kappa /p)}\right).$$

Therefore, for fixed values of *p* and *κ* the sign of the real part of the derivative can be evaluated for *Ω*_1−4_, hence, from (2.9), for all associated values of $T_{1-4}^{n}$, $n\in \{0\}\cup Z^{+}$.

As an example, let us consider *p*=0.2 and *κ*=2. With these values, there exist four non-negative real solutions for (2.5); *Ω*_1_=2.78, *Ω*_2_=1.14, *Ω*_3_=3.50 and *Ω*_4_=0.91. The associated values of delays are shown in table 1 for *n*=0−3. In terms of stability, the sign of (2.13) is negative for *Ω*_1_ and *Ω*_4_, whereas it is positive for *Ω*_2_ and *Ω*_3_. This means that the stability of the equilibrium changes from unstable to stable for the corresponding values of $T_{1}^{n}$ and $T_{4}^{n}$ and from stable to unstable for $T_{2}^{n}$ and $T_{3}^{n}$.

**Table 1.** Values of critical delay for the corresponding critical frequencies considering the 2*π* periodicity of the phase margin (by increasing *n*).

	delay			
frequency	*n*=0	*n*=1	*n*=2	*n*=3
*Ω*_1_	1.13	3.39	5.685	7.92
*Ω*_2_	2.75	8.26	13.78	19.29
*Ω*_3_	0	1.80	3.60	5.40
*Ω*_4_	6.91	13.82	20.74	27.65

**Figure 3.**
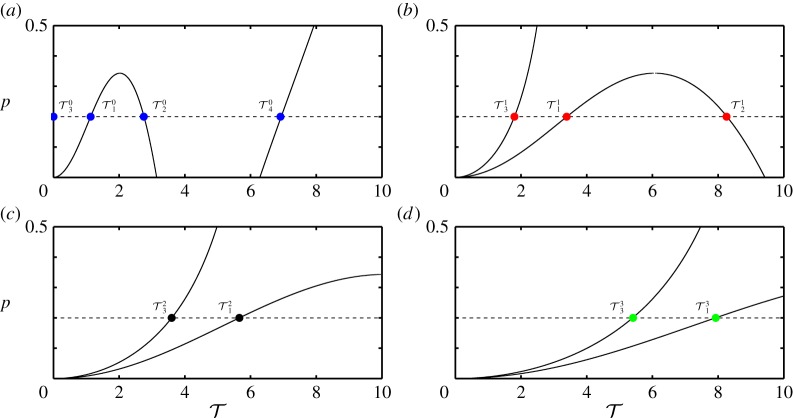
Two-parameter stability boundary curves in the (T,p)-plane for *n*=0 (*a*), *n*=1 (*b*), *n*=2 (*c*) and *n*=3 (*d*). The horizontal dashed line is at *p*=0.2 and the marked values of T denote the critical time delays where a switch in stability for the corresponding equilibrium occurs. (Online version in colour.)

This analysis may also be performed for other values of *p*. If, for a chosen number of *n*, the values of $T_{1-i}^{n}$—where *i* is the number of solutions for each value of *p*—are plotted for a continuous range of *p*, the respective limit points generate boundary curves in the (T,p)-plane. This is shown for *κ*=2 in figure 3*a*–*d*, where the boundary curves are presented for *n*=0,1,2,3, respectively. The horizontal dashed line in figure 3*a*–*d* is at *p*=0.2 and, hence, the marked intersections with the boundary curves correspond to the respective values in table 1.

**Figure 4.**
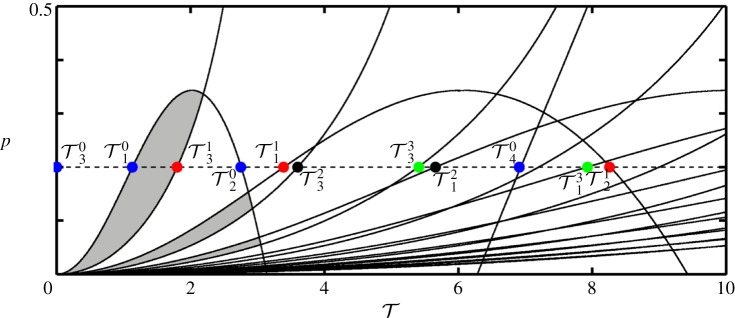
Two-parameter stability diagram of the substructured two degree-of-freedom system in the (T,p)-plane for *ζ*_*m*_=*ζ*_*μ*_=0. Shown are stability boundary curves (solid) and the line *p*=0.2 (dashed). There are infinitely many stability boundary curves as well as stable regions. They gradually approach the T-axis as n→∞. Only stability boundary curves that correspond to *n*=0,…,9 are shown. Shaded regions correspond to stable equilibria. (Online version in colour.)

As discussed, for all values of *n*, $T_{1}^{n}$ and $T_{4}^{n}$ mark values where the sign of the real part of the respective roots become negative, whereas $T_{2}^{n}$ and $T_{3}^{n}$ marks a change in the opposite direction. For evaluating the stability of the substructured system, stability switches for all *n* have to be considered together. The boundary curves for *n*=0,…,9 are shown in figure 4. The shaded regions correspond to parameter values for which all eigenvalues of (2.4) have negative real parts and, hence, the substructured system is asymptotically stable. Owing to the fact that only a finite number of the eigenvalues can be calculated, the stability diagram cannot be complete. However, larger values of *n* give larger eigenvalues which only affect the stability at small values of *p*. Note that, due to being undamped, the system is marginally stable for the case of zero delay, which is in agreement with the expected results for the system being emulated.

### (b) Frequency domain method

If either of the damping coefficients is non-zero, obtaining the roots of (2.5) analytically is difficult. Therefore, to study the qualitative changes of the stability caused by damping, a frequency domain analysis on the substructured system for the case of T=0 is performed and the stability is evaluated via Bode plots. The equivalence of the frequency and time domain analyses in terms of the resulting critical delays can be shown by first determining the critical values for the undamped case in the frequency domain and comparing the results with those of the time domain analysis. Therefore, first we analyse the undamped system, followed by the study of the damped system.

To begin with, the open-loop transfer function of the substructured system for the case of T=0 is derived. To this end, equations (2.1) are transformed into the frequency domain by the Laplace transform. The input to the physical substructure is the interface displacement from the numerical model—*y*_*μ*_ in this case—and the output is the feedback force *f* (figure 1). With reference to figure 1, the transfer function from the interface displacement to the interface force for the physical substructure is given by

$$G_{p}(\hat{s})=\frac{\hat{F}}{Y_{\mu }}=\frac{p{\hat{s}}^{2}(2\zeta_{m}\hat{s}+\kappa )}{p{\hat{s}}^{2}+2\zeta_{m}\hat{s}+\kappa },$$

where $\hat{s}=s/\omega_{n}$ is the normalized Laplace-operator and $\hat{F}=F/\mu \omega_{n}^{2}$. Similarly, the interface force (with opposite sign) to interface displacement transfer function for the numerical substructure reads

$$G_{n}(\hat{s})=\frac{Y_{\mu }}{\left(-\hat{F}\right)}=\frac{1}{{\hat{s}}^{2}+2\zeta_{\mu }\hat{s}+1}.$$

In order to simplify the notation, the *hat* is removed from the subsequent expressions. The open-loop transfer function for the non-dimensionalized system for the case of zero delay, therefore, is given by *G*(*s*)=*G*_n_(*s*) *G*_p_(*s*). It reads

2.14 $$G(s)=\frac{2\zeta_{m}ps^{3}+\kappa ps^{2}}{ps^{4}+2(\zeta_{m}+p\zeta_{\mu })s^{3}+(\kappa +p+4\zeta_{m}\zeta_{\mu })s^{2}+2(\zeta_{\mu }\kappa +\zeta_{m})s+\kappa }.$$

#### (i) Stability analysis in the frequency domain

**Figure 5.**
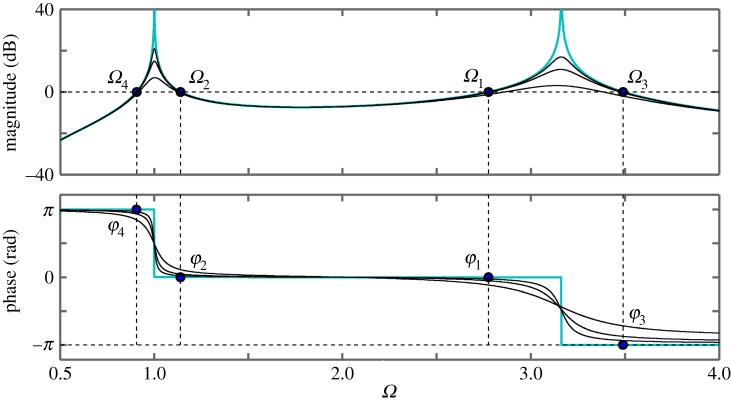
Bode plot of the transfer function *G*(*s*) of (2.14) for *κ*=2, *p*=0.2 and for different cases of damping: (*ζ*_*m*_;*ζ*_*μ*_)=(0.01; 0.01), (*ζ*_*m*_; *ζ*_*μ*_)= (0.02; 0.02) and (*ζ*_*m*_; *ζ*_*μ*_)=(0.05; 0.05) (black curves). The case of (*ζ*_*m*_; *ζ*_*μ*_)=(0; 0) is also shown for reference (blue/light curves).The dots in the magnitude plot represent the crossover frequencies. The dots in the phase plot represent the phase angles corresponding to the crossover frequencies. (Online version in colour.)

First, we study the undamped system by means of the Bode plot of (2.14). In order for the results to be comparable with those of the time domain analysis, we use the same parameters for the evaluation; *p*=0.2 and *κ*=2. The Bode plot is shown in figure 5, where the blue/light curves represent the undamped case and the black curves represent the cases of (*ζ*_*m*_,*ζ*_*μ*_)=(0.01,0.01),(0.02,0.02),(0.05,0.05).

For (2.14), there exist two pairs of complex conjugate poles, which, for the case of zero damping, are given by $P_{1,\;2}=\pm \sqrt{\kappa /p}\;i$ and *P*_3, 4_=±*i*. The magnitudes of the poles represent the non-dimensional natural frequencies of the physical and numerical parts, respectively. For the chosen values of *p* and *κ*, these frequencies are *Ω*_ph_=3.16 and *Ω*_n_=1, which correspond to the peaks in the magnitude plot. Further, there exist four crossover frequencies, *Ω*_1−4_ where the gain of (2.14) is unity; the associated phase angles are denoted by *φ*_1−4_. Note, that in the case of zero damping, the crossover frequencies are the same as those obtained as the solutions of (2.7).

To evaluate how the interface delay affects the stability, instead of introducing the delay directly via the transfer function, the phase margins at the crossover frequencies are considered. The phase margin is defined as the difference of the phase angle from −*π* at the corresponding crossover frequencies. Moreover, the phase margin is directly related to the critical delay $T_{1-4}$. Taking into account the 2*π* periodicity of the phase angle, the relationship between the phase angle and the associated critical delay is given by

2.15 $$T_{j}^{n}=\frac{\varphi_{j}+(2n+1)\pi }{\Omega_{j}},\;n\in \{0\}\cup Z^{+},$$

where *j*=1,…,*m* and *m* is the number of crossover frequencies for the given parameters. For example, in the undamped case, for *n*=0 the phase angles are *φ*_1,2_=0, *φ*_3_=−*π* and *φ*_4_=*π* and the corresponding delays are $T_{1}^{0}=1.13$, $T_{2}^{0}=2.75$, $T_{3}^{0}=0$ and $T_{4}^{0}=6.91$. These are the same values as the respective values in table 1. It can be shown that, if $T_{j}^{n}$ are determined for a continuous range of *p*, one obtains the same stability curves in the (T,p)-plane as presented in figure 4.

The next step is to determine how the stability changes at the critical delays. To this end, we apply the Nyquist criterion and study how that relates to the Bode plot. According to the Nyquist criterion *Z*=*P*−*N*, that is, the number of closed-loop poles on the right-hand side of the complex plane, *Z*, is equal to the difference between the number of open-loop poles on the right-hand side of the complex plane, *P*, and the number of encirclements, *N*, of the −1+0*i* point. Note that when the delay is zero, the closed-loop system is stable due to the fact that the open-loop system is stable by nature, *P*=0, and the closed-loop system exactly replicates the system being emulated, therefore *Z*=*N*=0.

As T increases and passes through a curve in the stability map, the loop gain on the Nyquist plot crosses the −1+0*i* point at the corresponding crossover frequency. If this crossing is an entry into the unit circle as frequency increases, the number of encirclements, hence the number of closed-loop poles on the right-hand side of the complex plane, increases by two; one pair of complex poles are destabilized. On the other hand, if at the crossover frequency the loop gain leaves the unit circle, the number of encirclements, hence the number of closed-loop poles reduces by two. This restabilizes a pair of complex poles. Here, we note that if the gain of the transfer function is greater than unity as Ω→∞, then the Nyquist plot has infinitely many encirclements for any T>0 becomes greater than zero. This means that, even though poles may be restabilized as T increases, the system may never recover from instability. An example of this will be shown in §3.

In terms of the Bode plot, for this example, the unit circle crossing corresponds to the crossover frequencies *Ω*_1−4_ and entering (leaving) the unit circle is associated with negative (positive) gradients of the magnitude plot. Therefore, at $T_{2,3}^{n}$, a pair of poles are destabilized and at $T_{1,4}^{n}$ a pair of poles are restabilized with the system being asymptotically stable only when all the poles are stable. Considering figure 4 at *p*=0.2 as the delay increases from zero to $T_{3}^{0}$ destabilizes a pole-pair, which is then restabilized at $T_{1}^{0}$. Further, a pole-pair is destabilized at $T_{3}^{1}$, etc.

Now let us consider an example case with damping. According to figure 5, applying damping changes the crossover frequencies as well as the associated phase margins. In particular, when increasing *ζ*_*μ*_ the phase margin decreases at the frequency *Ω*_4_ and increases at the frequency *Ω*_2_. Further, when increasing *ζ*_*m*_ the phase margin decreases at the frequency *Ω*_1_ and increases at the crossover frequency *Ω*_3_. The gradients of the magnitude plot at the crossover frequencies do not change. This means that, for the given parameters, the asymptotically stable region becomes larger. If, again, the limit points are calculated for a range of *p*, the stability map in the (T,p)-plane can be generated for cases with damping.

**Figure 6.**
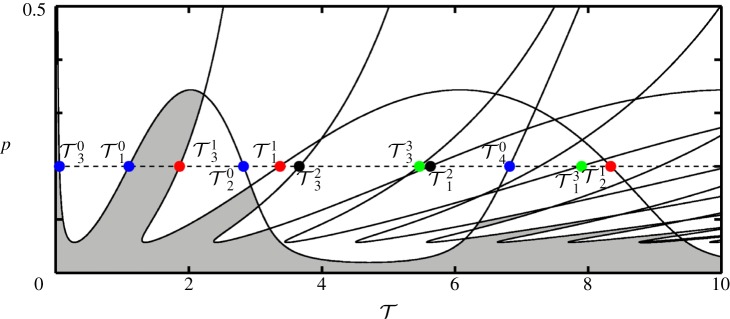
Two-parameter stability diagram of the substructured two degree-of-freedom model in the (T, p)-plane for (*ζ*_*m*_;*ζ*_*μ*_)=(0.01;0.01). Shown are stability boundary curves that correspond to *n*=0,…,9 (solid) and the line *p*=0.2 (dashed). Shaded regions correspond to stable equilibria. (Online version in colour.)

Figure 6 shows an example of the generated stability diagram for *κ*=2 and (*ζ*_*m*_; *ζ*_*μ*_)=(0.01; 0.01). The solid curves represent the stability boundaries and the dashed line denotes the horizontal section at *p*=0.2. The time delays, $T_{1-4}^{n}$ for *n*=1,2,3,4 with *p*=0.2, are labelled. The shaded regions correspond to stable equilibria.

Let us compare figure 4 to figure 6. It can be seen that, in general, adding damping to the system results in an increase of the overall region of stability. In terms of the phase margins of the system this means that, even though damping either increases or decreases the allowable phase shift until a sign change from negative to positive for certain eigenvalues occurs, the stability boundaries move in the direction of increased stability. Further, there exist two local minima in terms of the value of *p* for each boundary curve as T increases. The loci of these minima are determined by the damping coefficients. The first local minimum—the value of *p* for which $T_{1}^{n}=T_{3}^{n}$—depends mainly on the value of *ζ*_*m*_ and the second local minimum—the value of *p* for which $T_{2}^{n}=T_{4}^{n}$—depends mainly on the value *ζ*_*μ*_. The analytical derivation of the locus of the local minima is difficult due to the high order of the characteristic equation. Because only the qualitative change that damping has on the structure of the stability diagram is of interest, these calculations are not performed. It is worth noting that, even though stability may be maintained for larger delays as well with the addition of damping, simulations of the response of the system reveal that, for high values of delay, there is a significant loss of accuracy in the emulation of the original system dynamics.

Also note that analytical solutions or transfer functions are generally not available for more complex models with delay. For higher-dimensional systems, the determination of the stability boundaries may only be possible by means of numerical methods. In [21], for example, a nonlinear nose landing gear–fuselage system, that is based on the model of §2, is studied where the governing equations are functional differential equations of neutral type. These types of equations can be handled by the numerical continuation software Knut (previously known as PDDE-CONT) [22]. Further applications of the software can be found, for example, in [23,24].

## 3. The effect of changing the interface

**Figure 7.**
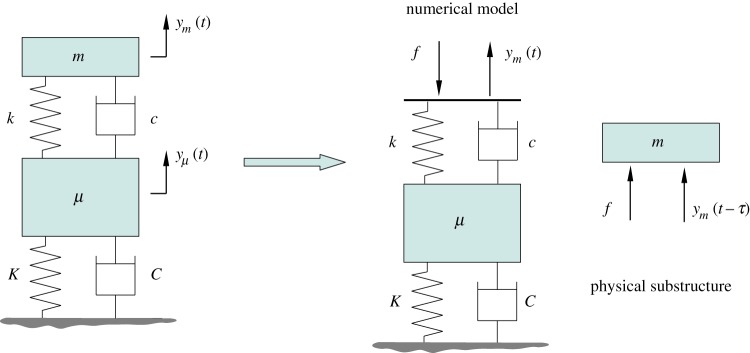
Schematic of the alternative way of substructuring the two degree-of-freedom model. The numerical part consists of the mass–spring–damper unit fixed to the ground and the spring and damper of the mass–spring–damper unit on the top of the structure. The physical substructure is the mass *m* only. (Online version in colour.)

Let us consider a different location for the numerical–physical interface in the two degree-of-freedom mass–spring–damper system. Here, the interface is right under the mass of the top oscillator instead of at the attachment points of its spring and damper. The schematic of this arrangement is shown in figure 7. The equations of motion for the original, non-substructured system are given by (2.1). In this configuration, the displacement of the mass *m* is delayed compared to the displacement of the endpoints of the spring and damper (figure 7). In terms of the non-dimensional equations of motion, this means that the displacement *y*_*m*_ and its derivatives in the terms corresponding to the physical part are delayed with the non-dimensional time delay $T=\omega_{n}\tau$. Therefore, the mathematical model of the substructured system reads

3.1 $$\begin{array}{rl} & y_{\mu }^{\prime \prime }(T)+2\zeta_{\mu }y_{\mu }^{\prime }(T)+y_{\mu }(T)-2\zeta_{m}(y_{m}^{\prime }(T)-y_{\mu }^{\prime }(T))-\kappa (y_{m}(T)-y_{\mu }(T))=0\\ \;\text{and}\; & py_{m}^{\prime \prime }(T-T)+2\zeta_{m}(y_{m}^{\prime }(T)-y_{\mu }^{\prime }(T))+\kappa (y_{m}(T)-y_{\mu }(T))=0. \end{array}\}$$

Here, only the inertia term of the mass *m* is delayed. Equation (3.1)_1_ is an ordinary differential equation and (3.1)_2_ is a functional differential equation of advanced type (AFDE), because only the highest derivative appears with a delayed argument. By adding T to all the arguments in (3.1)_2_, a simple shift in time is applied and the equation can be rewritten as

3.2 $$py_{m}^{\prime \prime }(T)=-2\zeta_{m}(y_{m}^{\prime }(T+T)-y_{\mu }^{\prime }(T+T))-\kappa (y_{m}(T+T)-y_{\mu }(T+T)).$$

The above equation reveals why this type of differential equation is called ‘advanced’: the current value of the derivative of some state—*y*_*m*_′ in this case—is determined by the future (advanced) values of the state. In-depth studies on AFDEs are limited. Some basic properties are discussed in, for example, [25] and a practical application is shown in [26]. Unlike linear retarded functional differential equations that have infinitely many characteristic roots on the left-hand side of the complex plane, linear AFDEs have infinitely many characteristic roots on the right-hand side of the complex plane and are, therefore, strongly or infinitely unstable [26]. Nevertheless, in order to demonstrate the delay margin method and compare the results with the case discussed in §2, we present both the time- and frequency domain analyses of this system.

### (a) Analytical time domain analysis

By converting (3.1)_1_ and (3.2) to a vector equation in the form of ${\text{x}}^{\prime }(T)=\hat{\text{A}}\text{x}(T)+\hat{\text{B}}\text{x}(T+T)$, the stability of the system can be studied. For the undamped case, the expressions for the pure imaginary eigenvalues of the characteristic equation are given by

3.3 $$\begin{array}{rl} \Omega_{1,2}^{2} & =\frac{1}{2}\left(\kappa \left(1-\frac{1}{p}\right)+1\right)\mp \frac{1}{2}\sqrt{{\left(\kappa +1\right)}^{2}-\frac{2\kappa }{p}(\kappa -1)+\frac{\kappa^{2}}{p^{2}}}\\ \;\text{and}\;\Omega_{3,4}^{2} & =\frac{1}{2}\left(\kappa \left(1+\frac{1}{p}\right)+1\right)\pm \frac{1}{2}\sqrt{{\left(\kappa +1\right)}^{2}+\frac{2\kappa }{p}(\kappa -1)+\frac{\kappa^{2}}{p^{2}}}. \end{array}\}$$

The two roots defined by (3.3)_2_ are the same as the roots of the respective polynomial of the original structure, see (2.7). However, *Ω*_1_ and *Ω*_2_ are different. Here, $\Omega_{1}^{2}$ is always negative for positive values of *κ* and *p* and, hence, *Ω*_1_ is always imaginary. Consequently, it is not a suitable solution, meaning that, in this substructuring setup, there are only three critical eigenvalues, *Ω*_2−4_, and, hence, associated critical delays $T_{2-4}^{n},\;n\in \{0\}\cup Z^{+}$.

Moreover, the gradient analysis—which is performed in the same manner as discussed in §2—shows that for the chosen fixed values of *κ*=2 and *p*=0.2 the sign is negative for *Ω*_3_ and *Ω*_4_, whereas it is positive for *Ω*_2_. Therefore, the stability of the equilibrium changes from unstable to stable for the corresponding values of $T_{3}^{n}$ and $T_{4}^{n}$, whereas from stable to unstable for $T_{2}^{n}$. This means that $T_{2}^{n}$ and $T_{4}^{n}$ mark the same change in stability as in the first substructuring case. However, $T_{3}^{n}$ has the opposite effect; the corresponding root is stable *until* it passes the stability boundary curve (whereas in the other case, it only becomes stable when the limit point is passed).

**Figure 8.**
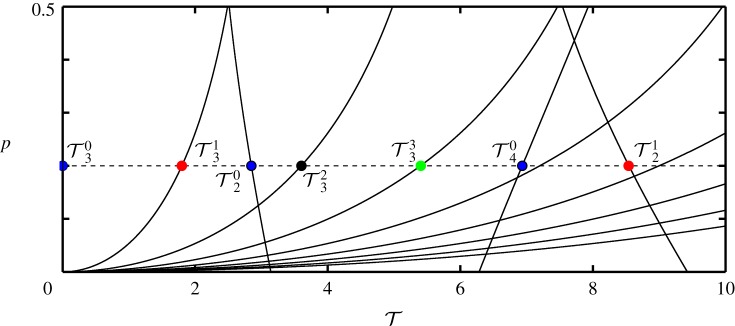
Stability of the equilibrium of the substructured two degree-of-freedom model with an alternative position of the interface in the (T,p)-plane for *ζ*_*m*_=*ζ*_*μ*_=0. There are infinitely many stability boundary curves as well as stable regions. They gradually approach the T-axis as n→∞. Shown are the stability boundary curves that correspond to *n*=0,…,7. For all values of parameters *p* and T the system is unstable. (Online version in colour.)

The stability boundary curves for *n*=0−8 with *κ*=2 are shown in figure 8. Horizontal sections of the diagram correspond to fixed values of *p*. The dashed line is at *p*=0.2 and the marked values correspond to $T_{2-4}^{n}$ for *n*=0,1,2,3. Here, for each value of *n* all parameters that are to the left of the $T_{3}^{n}$ boundary curves result in positive eigenvalues. Owing to the fact, that n→∞, this configuration is ultimately unstable. This clearly demonstrates that by shifting the interface position within the structure the stability can be changed significantly.

### (b) Frequency domain analysis

When damping is considered, the behaviour can be studied in the frequency domain just as for the substructuring case in §2. Here, the input to the physical substructure is the displacement *y*_*m*_ instead of *y*_*μ*_. Owing to the change in the interface, the transfer function between the interface displacement and interface force of the physical substructure is now given by

$$G_{p}(s)=\frac{F}{Y_{m}}=ps^{2},$$

whereas the transfer function between the interface force (with opposite sign) and the interface displacement of the numerical part reads

$$G_{n}(s)=\frac{Y_{m}}{\left(-F\right)}=\frac{s^{2}+(\zeta_{m}+\zeta_{\mu })s+\kappa +1}{\left(2\zeta_{m}s+\kappa )(s^{2}+2\zeta_{\mu }s+1\right)}.$$

Therefore, the open-loop transfer function *G*(*s*)=*G*_n_(*s*) *G*_p_(*s*) is given by

3.4 $$G(s)=p\frac{s^{4}+2(\zeta_{m}+\zeta_{\mu })s^{3}+(\kappa +1)s^{2}}{2\zeta_{m}s^{3}+(\kappa +4\zeta_{m}\zeta_{\mu })s^{2}+2(\zeta_{\mu }\kappa +\zeta_{m})s+\kappa }.$$

**Figure 9.**
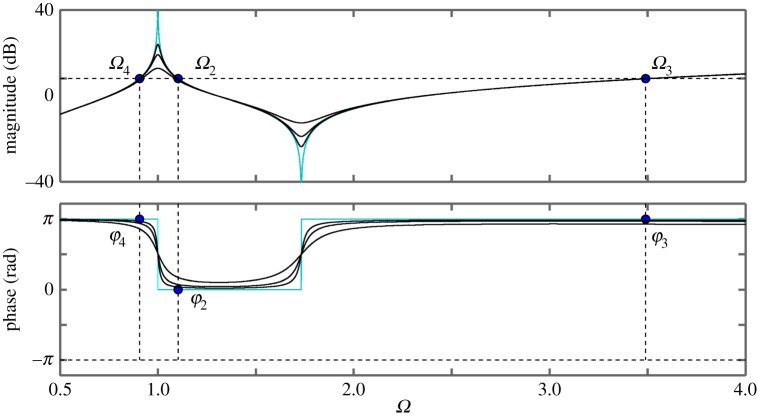
Bode plot of the transfer function *G*(*s*) of (3.4) with the alternative position of the interface for *κ*=2, *p*=0.2 and for different cases of damping: (*ζ*_*m*_; *ζ*_*μ*_)=(0.01; 0.01), (*ζ*_*m*_; *ζ*_*μ*_)= (0.02; 0.02) and (*ζ*_*m*_; *ζ*_*μ*_)=(0.05; 0.05) (black curves). The case of (*ζ*_*m*_; *ζ*_*μ*_)=(0; 0) is also shown for reference (blue/light curves). The dots in the magnitude plot represent the crossover frequencies. The dots in the phase plot represent the phase angles corresponding to thecrossover frequencies. (Online version in colour.)

Note, that the transfer functions are for the non-dimensionalized system; however, the *hat* has been removed again for simpler notation. The Bode plot for the case of *p*=0.2, *κ*=2 is shown in figure 9. Owing to the fact that (3.4) is an improper transfer function, G(s)→∞ as Ω→∞. The case of *ζ*_*m*_=*ζ*_*μ*_=0 is represented by the blue/light curves, whereas the black curves correspond to the cases of different values of damping ratio. The crossover frequencies for the undamped case are denoted by *Ω*_2−4_ and the phase angles by *φ*_2−4_.

The transfer function (3.4) has one pair of complex conjugate poles which, for the undamped case, are *P*_1,2_=±*i*. The magnitude of these correspond to the natural frequency of the numerical part. It is *Ω*_n_=1 and it is associated with the resonance peak in the magnitude plot. Further, there exists one pair of complex conjugate zeros which, for the undamped case, are given by $z_{1,2}=\pm \sqrt{\left(1+\kappa \right)}\;i$. For the chosen value of *κ*, this frequency is $\Omega_{n}^{*}=1.73$, which corresponds to the anti-resonance in the magnitude plot. The undamped crossover frequencies are *Ω*_2_=1.14, *Ω*_3_=3.492 and *Ω*_4_=0.909, whereas the corresponding phase angles are *φ*_2_=0 and *φ*_3,4_=*π*.

From the phase margins and the gradients of the magnitude plot at the crossover frequencies, the stability of the system can be evaluated. The positive gradient at *Ω*_4_ and *Ω*_3_ means that corresponding poles are stabilized, whereas the negative gradient at *Ω*_2_ means that poles are destabilized. However, owing to the fact that the gain of (3.4) is greater than unity as Ω→∞, the Nyquist plot has infinitely many encirclements as T becomes greater than zero. This means that, even though poles are restabilized as T increases, the system never recovers from instability.

Further, according to figure 9, damping does not change the crossover frequencies significantly. However, it alters the respective phases and, therefore, changes the critical values of delay. Yet, this does not change the overall stability. Further, the system remains unstable for all values of *p*, *ζ*_*m*_ and *ζ*_*μ*_. This is due to the fact that as *p* approaches zero, the frequency *Ω*_3_ approaches infinity, see (3.3). In turn, the respective time delay approaches zero. Therefore, for all considered phase angles, the respective stability boundary curves cross the (T; p)=(0;0) point. This suggests that, even though the stability boundary curves change slightly due to damping, the region of instability remains the entire (T,p)-plane. This means that, even if damping is present, system (3.1) is nonetheless unconditionally unstable. This, again, is in accordance with the unstable nature of advanced type functional differential equations.

Here, we remark that considering a constant, state-independent delay is only reasonable over the bandwidth of the actuator. This means that in reality some regions might be stable in the substructuring case too, given that above the bandwidth the gain is less than unity.

The examples of the two degree-of-freedom system show that a change in the interface location can significantly alter the stability of the substructuring experiment. Moreover, they also reveal that compliance between the two masses, on either side of the interface, reduces the region of stability in terms of the parameters *p* and T, compared to the limiting case of κ→∞, that is, of the split mass system, studied in [16].

**Figure 10.**
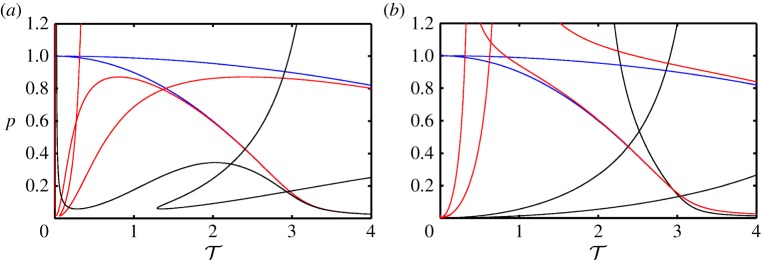
Stability boundary curves for the substructuring cases of §2 (*a*) and of §3 (*b*) with κ→∞ (blue), *κ*=200 (red) and thenominal value *κ*=2 (black). (Online version in colour.)

This is illustrated in figure 10. Figure 10*a* shows boundary curves in the (T,p)-plane for the substructuring case of §2 with κ→∞ (blue), *κ*=200 (red) and the nominal value *κ*=2 (black). Figure 10*b* shows these curves for the substructuring case of §3. The blue curves, κ→∞, are identical in figure 10*a*,*b* as the limiting case the systems are identical to the split mass system in [16]. However, as shown in figure 10*a*, the larger the compliance between the masses on the physical side, the smaller the maximum value of *p* above which the system is unstable regardless of T. The region of stability is also affected by the ‘intersecting’ unstable regions. On the other hand, when the compliance becomes greater than zero on the numerical side, infinitely many curves, corresponding to the destabilizations of pole-pairs, appear in the positive T direction. The analysis of the two different substructuring cases, therefore, shows that the split mass system is the preferred solution for a substructuring test. If there is compliance either side of the interface, the stable region of parameters either becomes significantly reduced or completely disappears.

## 4. Substructuring a cable

Having studied this conceptual two degree-of-freedom oscillator, let us now extend the study of substructurability to a higher-dimensional system with continuously distributed mass. In this section, we study the effect of the location of the physical–numerical interface on a clamped cable by means of the delay margin approach. Note, that a similar problem is studied in [27], where the authors consider an elastic beam with delayed feedback control. Although the methods used here are different, the effect of the delay on the stability of the substructured cable is qualitatively comparable with that on the beam system in [27].

The equation that describes the dynamics of the vibrating continuous cable defined in the *x*-direction reads

4.1 $$\frac{\partial^{2}u}{\partial t^{2}}=\frac{T}{\varrho A}\left(\frac{\partial^{2}u}{\partial x^{2}}+\gamma \frac{\partial^{2}u}{\partial t\partial x^{2}}\right),$$

where, *A*, *l* and *ϱ* are the cross-sectional area, length and material density of the cable, respectively, *T* is the tension in the cable and *u* is the vertical displacement along the cable. The structural damping is considered to be proportional to the stiffness with a coefficient *γ*; for more information on modelling cable dynamics, see e.g. [28].

To analyse substructurability, here we use a finite-element discretization of a cable, which is clamped at both ends, with the total number of elements being *N*_tot_. The endpoints of an element are *x*_*j*_ and *x*_*j*+1_, whereas the vertical displacements of those are *u*_*j*_ and *u*_*j*+1_, where *j*=1,2,…,*N*_tot_+1. The mass and stiffness matrices of a linear two-dimensional cable element are given by

$$\text{M}=\frac{\varrho Al}{6N_{\mathrm{tot}}}\left(\begin{array}{cc} 2 & 1\\ 1 & 2 \end{array}\right)\;\mathrm{and}\;\text{K}=\frac{TN_{\mathrm{tot}}}{l}\;\left(\begin{array}{cc} 1 & -1\\ -1 & 1 \end{array}\right).$$

When the cable is split for substructuring, out of the *N*_tot_ elements *N*_p_ elements comprise the physical part; this results in *N*_n_=*N*_tot_−*N*_p_ elements for the numerical part. The location of the interface is at *x*_*N*_p_+1_. The constraint force that acts at the interface and, hence, couples the subsystems is *f*. This force corresponds to the vertical component of the tension force in the original cable, hence, it is proportional to the derivative with respect to the location of the curve described by the cable.

Considering the first natural frequency of the undamped cable, which is given by

$$\omega_{1}=\frac{\pi }{l}\sqrt{\frac{T}{\varrho A}},$$

the equations of motion may be given in a non-dimensionalized form with respect to *ω*_1_. For the physical part, the equation in the frequency domain reads

4.2 $$({\bar{\text{M}}}_{\text{p}}\;{\hat{s}}^{2}+a(\gamma \hat{s}+1){\bar{\text{K}}}_{\text{p}})\left(\begin{array}{c} u_{2}\\ \vdots \\ u_{N_{p}+1} \end{array}\right)=\left(\begin{array}{c} 0\\ \vdots \\ \;\;\hat{f} \end{array}\right),$$

and for the numerical part is given by

4.3 $$({\bar{\text{M}}}_{\text{n}}\;{\hat{s}}^{2}+a(\gamma \hat{s}+1){\bar{\text{K}}}_{\text{n}})\left(\begin{array}{c} u_{N_{p}+1}\\ \vdots \\ u_{N_{\mathrm{tot}}} \end{array}\right)=\left(\begin{array}{c} -\hat{f}\\ \vdots \\ 0 \end{array}\right).$$

Here, $\hat{s}=s/\omega_{1}$ is the normalized Laplace-operator, *a*=6*N*^2^_tot_/*π*^2^ and $\hat{f}=(6N_{\mathrm{tot}}l/\pi^{2}T)f$ and *u*_*j*_ is the vertical displacement of the corresponding point of an element. The matrices ${\bar{\text{M}}}_{\text{p}}$ and ${\bar{\text{K}}}_{\text{p}}$ are of size *N*_p_×*N*_p_ and the matrices ${\bar{\text{M}}}_{\text{n}}$ and ${\bar{\text{K}}}_{\text{n}}$ are of size *N*_n_×*N*_n_ with all elements, except for the leading, sub- and super-diagonals, being zero. For both mass (stiffness) matrices, the sub- and super-diagonal elements are all 1 (−1) and the leading diagonal elements are all 4 (2) with the exemption of the *last* leading diagonal element of ${\bar{\text{M}}}_{\text{n}}$ $\left({\bar{\text{K}}}_{\text{n}}\right)$ and the *first* leading diagonal element of ${\bar{\text{M}}}_{\text{p}}$ $\left({\bar{\text{K}}}_{\text{p}}\right)$, which are 2 (1).

Owing to the cable being clamped at both ends, the vertical displacements of endpoints *x*_1_ and *x*_*N*_tot_+1_ do not appear in the dynamic equations as they are zero for all times. The respective static equations give the relationship between the constraint force at the clamp and the displacement of the other end of the element.

### (a) Force–displacement transfer functions

In order to study the effect of the transport delay occurring at the interface, the phase margin of the open-loop transfer function of the coupled physical–numerical system is considered. To this end, the transfer functions of both the physical and numerical parts in terms of the force and displacement at the interface are derived. From (4.2), the transfer function of an arbitrary element of the physical part, in terms of the displacements of its endpoints (again, the *hat* is removed from all subsequent $\hat{s}$ and $\hat{F}$ for simplicity), can be written as

$$T_{j,j+1}=\frac{U_{j}}{U_{j+1}}=-\frac{s^{2}-a(\gamma s+1)}{\left(s^{2}-a(\gamma s+1))T_{j-1,j}+4s^{2}+2a(\gamma s+1\right)},\;j=1,2,\ldots ,N_{p}.$$

Owing to the coupling between the elements, the transfer function of the *j*th element can be given from the transfer function of the *j*−1th element. Note, that the transfer function *T*_1,2_ for the first element may be set to zero as the displacement *U*_1_=0. Furthermore, at the physical side of the interface the input is the displacement from the numerical part, *U*_*N*_p_+1_ and the output is the force *F*. Therefore, the transfer function reads

4.4 $$G_{p}(s)=\frac{F}{U_{N_{p}+1}}=(s^{2}-a(\gamma s+1))T_{N_{p},N_{p}+1}+2s^{2}+a(\gamma s+1).$$

The transfer function of an arbitrary element of the numerical part can be derived from (4.3) in the same manner. This time the other end of the cable is considered first. Starting from the clamped *N*_tot_th element, the transfer function of each neighbouring element is given by

$$\begin{array}{rl} T_{j,j-1} & =\frac{U_{j}}{U_{j-1}}=-\frac{s^{2}-a(\gamma s+1)}{\left(s^{2}-a(\gamma s+1))T_{j+1,j}+4s^{2}+2a(\gamma s+1\right)},\\ & \;j=N_{\mathrm{tot}}+1,N_{\mathrm{tot}},\ldots ,N_{p}+2, \end{array}$$

hence, the transfer function of the *j*th element can be given from the transfer function of the *j*+1th element. The transfer function for the last element *T*_*N*_tot_+1,*N*_tot__ may be set to zero as *U*_*N*_tot_+1_=0. The transfer function for the numerical side of the interface is defined by the force from the physical part, −*F*, Being the input and the calculated displacement, *U*_*N*_p_+1_, as the output. It reads

4.5 $$G_{n}(s)=\frac{U_{N_{p}+1}}{\left(-F\right)}=\frac{1}{\left(s^{2}-a(\gamma s+1))T_{N_{p}+2,N_{p}+1}+2s^{2}+a(\gamma s+1\right)}.$$

Therefore, from (4.4) and (4.5) the open-loop transfer function of the coupled physical–numerical model is given by

4.6 $$G(s)=G_{p}(s)G_{n}(s)=\frac{\left(s^{2}-a(\gamma s+1))T_{N_{p},N_{p}+1}+2s^{2}+a(\gamma s+1\right)}{\left(s^{2}-a(\gamma s+1))T_{N_{p}+2,N_{p}+1}+2s^{2}+a(\gamma s+1\right)}.$$

### (b) Critical delay and the mode shapes of unstable oscillations

**Figure 11.**
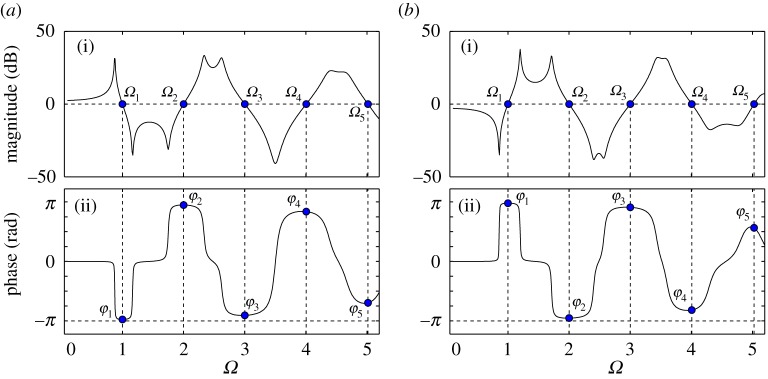
Bode-plots for the interface force–displacement transfer function *G*(*s*) of substructured cable for interface locations *L*_*i*_=0.43 (*a*) and *L*_*i*_=0.59 (*b*). The horizontal axis is the non-dimensionalized frequency. The *dots* denote the crossover frequencies and the corresponding phase angles. (Online version in colour.)

Here we use the delay margin approach to evaluate how the location of the interface along the cable affects the tolerable delay threshold, passing of which means the substructuring experiment becomes unstable. Example Bode plots for two different interface locations for a cable that is modelled with *N*_tot_=84 elements and *γ*=0.01, are shown in figure 11. Note, that due to the recursive formulation of (4.6), the transfer function may be of very high order. Indeed, it was found that *N*_tot_=84 is the maximum number of cable elements that could be handled by the relevant Matlab routines; nevertheless, this number of elements represents converged solutions.

Let us define *L*_*i*_=*N*_p_/*N*_tot_, that is, the ratio of the number of elements in the physical substructure with respect to the total number of elements. Therefore, *L*_*i*_ represents the length of the physical substructure, hence, the location of the physical–numerical interface with respect to the normalized length of the cable. The frequency response and associated phase angles for the case of *L*_*i*_=0.43 are shown in figure 11*a* and those for the case of *L*_*i*_=0.59 in figure 11*b*. The dots in figure 11 denote the crossover frequencies *Ω*_1−5_ and the associated phase angles *φ*_1−5_. As can be seen in figure 11, the crossover frequencies are in very close vicinity of the non-dimensionalized natural frequencies of the cable; the values of frequencies, associated phase and delay margins as well as the effect of passing the limit point on the stability are shown in table 2. The stabilizing/destabilizing property of the limit points are evaluated by the gradient of the magnitude plot at the crossover frequencies; positive (negative) gradient means that passing the limit results in a pair of eigenvalues crossing the imaginary axis from right to left (left to right) and, hence, the equilibrium being stabilized (destabilized). Here, we note that the non-dimensional delay margin values $T_{1-5}^{n}$ of table 2 are only given for *n*=0, even though there exist an infinite number of them just as with the two degree-of-freedom model in §§2 and 3. For the cable, however, due to the high number of destabilizing crossover frequencies, the stability is already determined by the principal values, *n*=0, of delay margin.

**Table 2.** Values of critical frequencies and corresponding critical delays when the length of the physical substructure is *L*_*i*_=0.43 or *L*_*i*_=0.59 of the full length. DS denotes destabilizing and *S* denotes stabilizing limit points. Bold marks the smallest destabilizinglimits.

interface location	0.43					0.59				
frequency	1.00	2.00	3.00	4.01	5.02	1.00	2.00	3.00	4.01	5.03
phase margin [rad]	0.072	6.123	0.289	5.774	0.953	6.220	0.144	6.000	0.617	4.568
delay margin	**0**.**072**	3.060	0.096	1.441	0.190	6.220	**0**.**072**	1.999	0.142	0.978
effect on stability	DS	*S*	DS	*S*	DS	*S*	DS	*S*	DS	*S*

The main difference between the two presented cases is that the smallest delay margin corresponds to the first natural frequency of the cable for the case of *L*_*i*_=0.43, whereas it is associated with the second natural frequency for the case of *L*_*i*_=0.59 (table 2). This is due to the fact that the smallest crossover frequency with a negative gradient, hence destabilizing effect, belongs to those natural frequencies. This means that if *L*_*i*_=0.43, once the system loses stability, the cable starts to oscillate at its first mode. On the other hand, if *L*_*i*_=0.59, the emerging oscillation corresponds to the second mode. This phenomenon reveals that not only does the location have an effect on the allowable delay in the system but it also influences the mode of vibration once stability is lost.

**Figure 12.**
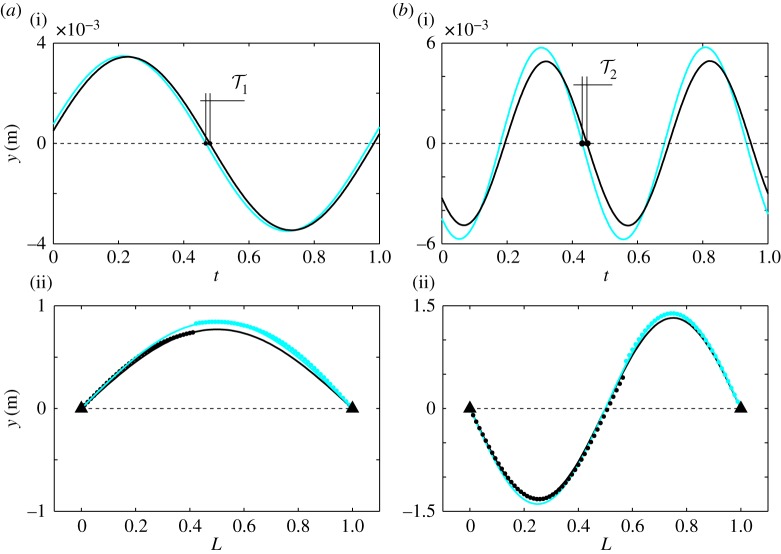
Time histories and vibration modes of the substructured cable in the unstable region for the cases of interface locations *L*_*i*_=0.43 (*a*) and *L*_*i*_=0.59 (*b*). Blue/light curves correspond to the numerical substructure and black curves correspond to the physical substructure. The displacements in (*a*(ii)) and (*b*(ii)) are enlarged in order for the discrepancy at the two sides of the interface to be visible. (Online version in colour.)

This is illustrated in figure 12. Here, time histories of both the numerical and physical sides of the interface as well as snapshots of the shape of the oscillating cable over the normalized length *L* are shown for the cases of the interface being at *L*_*i*_=0.43 in figure 12*a* and at *L*_*i*_=0.59 in figure 12*b*. The solution measure is the vertical displacement of the cable, *y*. Note that these interface locations are the same as for the Bode plots and delay margin values which are shown in figure 11 and table 2, respectively. Indeed, the delay values for the simulations are chosen to be close to but slightly higher than the threshold values suggested by table 2; namely, $T_{1}=0.075,$ for the case of *L*_*i*_=0.43 and $T_{2}=0.08$ for *L*_*i*_=0.59. Therefore, in both cases, the unstable oscillations of the substructured system can be studied. Figure 12*a*(i),*b*(i) shows the time histories and figure 12*a*(ii),*b*(ii) the mode shapes of the emerging unstable oscillations. In figure 12*a*(i),*b*(i), the blue/light curves represent the oscillation of the endpoint of the element at the numerical side of the interface, whereas the black curves represent the neighbouring point in the physical substructure (due to this, there is a slight difference in amplitude between the curves). Nonetheless, more importantly, it can be seen that the numerical side in both cases is ‘ahead’ of the physical substructure and the values of delay are $T_{1}$ and $T_{2}$, respectively. Further, the frequency of oscillation for the case of *L*_*i*_=0.59 is twice that of the case *L*_*i*_=0.43; these frequencies are the non-dimensional frequencies of *Ω*_2_=2 and *Ω*_1_=1. These results are also suggested by the delay margin calculations. Namely, for the case of *L*_*i*_=0.43 the minimum value of the delay margins corresponds to the first natural frequency of the original cable, whereas for the case of *L*_*i*_=0.59, it is associated with the second natural frequency.

The mode shapes of the oscillations are shown in figure 12*a*(ii),*b*(ii) in the (*L*,*y*)-plane. Here, the blue/light dots represent the displacements of the numerical side, whereas the black dots correspond to the displacements of the physical substructure; the vertical displacements are enlarged in order for the discrepancy in the displacements at the two sides of the interface (caused by the delay) to be visible. Furthermore, black and blue/light solid curves are fitted to the set of black and blue/slight dots, respectively. These curves are the shape functions of a cable clamped at both ends, that is: $A_{m}\sin(n\pi )$, where *A*_*m*_ is the maximum amplitude of the oscillation and *n*∈[1,2] for the presented cases. As can be seen, there is a slight deviation of the mode shape of the substructured system from that of the system being emulated; nevertheless, the difference is small enough for the model to be a good approximation.

### (c) Stability over the entire length of the cable

**Figure 13.**
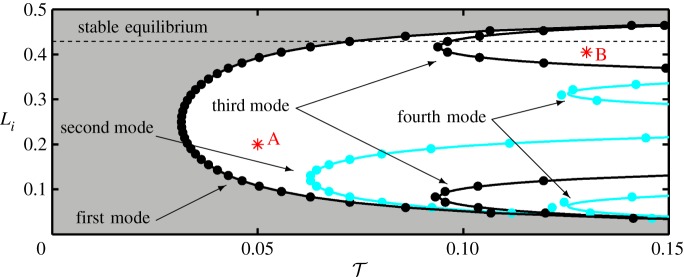
Two-dimensional stability map of the substructured cable in the $\left(T,L_{i}\right)$-plane for T∈[0,0.15] and *L*_*i*_∈[0,0.5]. The shaded area corresponds to the stable equilibrium solutions of the system. When the stability condition is violated, unstable oscillations emerge with well-defined modes of the emulated cable. Blue/light represent even numbered modes of the system and black is associated with odd numbered modes. The horizontal dashed line correspond to the case of *L*_*i*_=0.43. The points *A* and *B* show example locations for vibrations of first and third modes, respectively. (Online version in colour.)

The two example cases reveal the importance of the location of the interface in a substructuring experiment. However, in order to have a more global view on the influence of the location on the stability and on the vibration modes of the emerging oscillations, the delay margins must be calculated over the whole length of the cable for all possible locations allowed by the discrete number of elements. This is shown in figure 13 in the $\left(T,L_{i}\right)$-plane for T∈[0,0.3] and *L*_*i*_∈[0,0.5]. As expected, for each discrete value of *L*_*i*_ there are a number of delay margins in the plotted area which are associated with different natural frequencies. These are denoted by black and blue/light *dots* representing odd and even numbered modes, respectively. The horizontal dashed line represents the example case of *L*_*i*_=0.43. In order to approximate the cable as a continuum, continuous curves are generated based on the discrete values by interpolation. However, for the third and fourth modes a step change in *L*_*i*_ results in a large change in T and fewer points are available than for the first and second modes. This would lead to inaccurate results. Therefore, the symmetry of the cable (that is clamped at both ends) is used to overcome this difficulty. Namely, the second mode shape of the original cable can be imagined as two half length cables clamped at both ends. This is due to the mid-span zero-displacement node in the mode shape being equivalent to a clamp. Both cables are of half the length of the original full-length cable, they are connected at the node and oscillate in anti-phase (figure 12*b*(ii)). If the cable parameters do not change, halving the length implies doubled stiffness and, due to proportionality, doubled damping ratio as well. This means that the first natural frequency of a half-length cable is the same as the second natural frequency of the original full-length cable. Therefore, the delay margins associated with the first mode of this half-length cable also correspond to the second mode of the original with the same sufficient number of points for the interpolation. The same holds for higher modes as well. Hence, by using the delay margins calculated for first modes of reparametrized cables, continuous curves associated with higher modes can also be obtained.

The continuous curves in figure 13 are based on these calculations. The curve that connects the minima of the delay margins—associated with the first natural frequency of the original cable in the plotted region—forms the stability boundary of the system in terms of the interface location; the grey shaded area corresponds to the stable equilibrium solutions of the substructured system, where the oscillations caused by initial perturbation decay exponentially. There is some discrepancy between the generated curves and the original delay margin values denoted by the *dots*; these are most visible at the fourth mode. This is a result of deviations in the calculated phase margins as well as of the slightly erroneous location of the nodal points due to the finite number of elements being used and, hence, the mode shape as suggested by figure 12*b*(ii). Nevertheless, the curve that separates the stable and unstable regions associated with the first mode is not affected. In the region of instability different types of oscillations can be distinguished in the different enclosed areas. In general, for any fixed parameter pair, $\left(T,L_{i}\right)$, the frequency, hence the mode shape of the emerging oscillation, are those associated with the closest curve to the left of the chosen point. For example, the points *A* and *B*—marked by stars in figure 13—correspond to oscillations at the first mode and the third mode, respectively.

**Figure 14.**
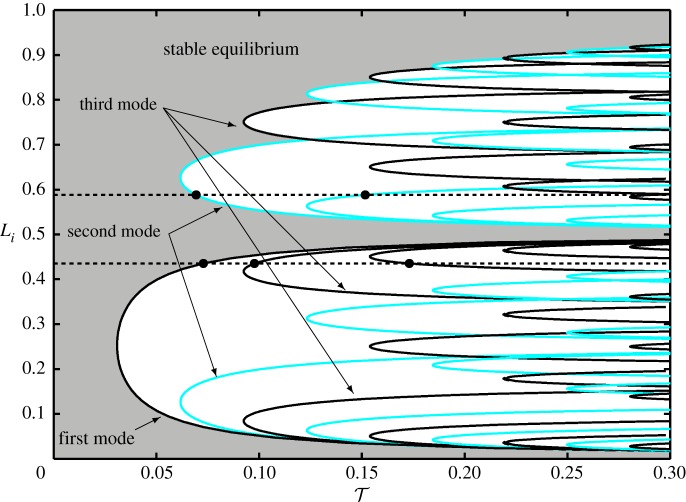
Two-dimensional stability map of the substructured cable in the $\left(T,L_{i}\right)$-plane for T∈[0,0.3] and *L*_*i*_∈[0,1]. The shaded area corresponds to stable equilibrium solutions of the system. When the stability condition is violated, unstable oscillations emerge with well-defined modes of theemulated cable. Blue/light represents even numbered modes and black is associated with odd numbered modes. The horizontal dashed lines correspond to the cases of *L*_*i*_=0.43 and *L*_*i*_=0.59. (Online version in colour.)

Let us now consider an extended stability diagram that covers the entire length of the substructured cable. It is shown in figure 14. Note, that due to the high number of modes not all the associated curves are shown. For the chosen range of T∈[0,0.3] only curves up to the ninth mode are present. As before, black curves correspond to odd number modes and blue/light curves are associated with even numbered modes. Again, the two horizontal dashed lines correspond to the example cases *L*_*i*_=0.43 and *L*_*i*_=0.59. As can be seen, in the interval of *L*_*i*_∈[0,0.5], that is, when the physical substructure is not longer than half of the cable, the stability boundary is defined by the curve corresponding to the first vibrational mode, with the minimum tolerance to delay being at *L*_*i*_=0.25; this is already shown in figure 13. This plot captures the substructurability of the cable system, with a stable response lost when the delay increases to T≈0.03 if the interface is at *L*_*i*_=0.25, whereas it is stable for any delay when *L*_*i*_=0.5. Considering the case of *L*_*i*_=0.5, the stability is due to the vertical component of the interface force always being zero in this case which decouples the numerical model from the physical substructure and, therefore, the delay at the interface has got no effect on the originally stable system. Moreover, *L*_*i*_=0.5 also marks the location of the mid-span zero-displacement node of the second vibration mode of the cable, the physical appearance of which has consequences for the stability in terms of the rest of the possible interface locations. Namely, a curve associated with some higher mode of the cable can only become a stability boundary of the substructured system if the appearance of the nodes related to the mode are not prohibited. For example, for the third mode to become the stability boundary, the physical substructure needs to be at least two-thirds of the cable; to allow the two associated nodes to appear.

### (d) The nonlinear cable

As already indicated in §1, a real-time dynamic substructuring test is generally performed on systems that are difficult to model numerically. This might be the case especially for nonlinear systems. Therefore, even though the linear study proved to be sufficient to explain the effect of the interface location on stability and system response, here, we briefly examine a nonlinear cable to further extend the results.

A significant source of nonlinearity in a taut cable is tension variation due to the dynamics of the cable, resulting in a dynamic time-dependent tension [28]. The partial differential equation that describes the cable vibrations with dynamic tension—to a second-order approximation—is given by

4.7 $$\frac{\partial^{2}u}{\partial t^{2}}=\frac{T_{d}}{\varrho A}\left(\frac{\partial^{2}u}{\partial x^{2}}+\gamma \frac{\partial^{2}u}{\partial t\partial x^{2}}\right)\;\mathrm{and}\;T_{d}=T\left(1+\frac{1}{2}{\left(\frac{\partial u}{\partial x}\right)}^{2}\right),$$

where *T*_*d*_ and *T* are the dynamic and static tension in the cable, respectively. Note, that if the dynamic tension is not considered, that is, if the nonlinear term in (4.7) is zero, then system (4.7) reduces to (4.1). When discretized along the length of the cable, this equation becomes a set of ordinary differential equations. Following the same non-dimensionalizing process as in §2, the equation describing the vertical motion of a general *j*th discretized point reads

$$u_{j}^{\prime \prime }=a\left(1+\frac{1}{2l_{e}^{2}}{\left(u_{j+1}-u_{j}\right)}^{2}\right)((u_{j+1}-2u_{j}+u_{j-1})+\gamma (u_{j+1}^{\prime }-2u_{j}^{\prime }+u_{j-1}^{\prime })),$$

where *l*_*e*_ is the distance between two adjacent discrete points along the cable and the *prime* refers to the differentiation with respect to the non-dimensional time *T*; see §2. When the numerical–physical interface is considered and the respective terms are changed to their delayed counterparts, the set of equations may be simulated for different values of non-dimensional delay, T, and interface location, *L*_*i*_. In this way, the change in the stability boundaries with respect to those of the linear system may be evaluated.

To understand the effects of including the nonlinearity, we note that two separate features need to be considered: the location of a stability boundary itself, and the response of the system once the stability boundary has been crossed. Nonlinear systems theory states that the stability of an equilibrium of a nonlinear system may be studied by that of the equivalent linear system. This is why the stability boundaries themselves are not notably affected by the inclusion of the dynamic tension, i.e. figure 14 is unaltered when the respective nonlinear terms are included. On the other hand, the nonlinearity is expected to have a discernible effect when a boundary curve is crossed and linear stability is lost. A study of the nonlinear system by means of simulation for initial parameter values near the stability boundaries in figure 14 showed that, once the respective stability boundary has been crossed, the response is indeed significantly different from that of the linear system. Taken together, this initial investigation provides a clear indication that the linear study presented here has value also in the nonlinear setting. A more detailed further exploration of nonlinear effects is, however, beyond the scope of this paper.

## 5. Conclusion

We studied the effect of the interface location on the stability of a real-time dynamic substructuring test. We used the term *substructurability* to refer to the feasibility of a stable experimental test in terms of the tolerance to transfer system delays at the interface between the numerical and experimental substructures. The effect of interface location on the substructurability was then considered. This was achieved by using two techniques. Firstly, analytical time domain analysis was discussed, which is convenient when there is no damping in the system. Then, for the more general damped case, where the analytical approach became impractical, stability boundaries were studied in the frequency domain. The crossover frequencies and the associated phase and delay margins were derived from Bode plots of the open-loop transfer function, and the stability of the system was evaluated directly from the Bode plots based on the Nyquist criterion.

To demonstrate these methods along with the concept of substructurability, firstly a two degree-of-freedom lumped mass–spring–damper system was studied for two different interface locations. This example allowed us to conclude that by changing the interface from one side of a spring to the other, the substructurability changes. Introducing compliance between the two masses in the physical substructure results in a conditionally substructurable system, where the stability of the experiment depends of the ratio of the two masses as well as the time delay in the transfer system. On the other hand, if the same compliance is in the numerical substructure, the system becomes ultimately unstable even in the presence of damping; therefore, the system is not substructurable with this configuration. Comparing our results to the split-mass reference model [16], we can also conclude that in terms of feasible substructuring tests, the split-mass system, where there is no compliance between the masses, is the preferred solution.

Then we applied the delay margin approach on the finite-element model of a cable that is clamped at both ends and evaluated the stability of the substructured system for all possible interface locations as given by the maximum feasible number of elements of the discretization. It was found that as opposed to the two degree-of-freedom model, the cable is substructurable for all possible interface locations; however, the tolerance to delay in each case is different. Moreover, not only does a change in the interface change the tolerance of the system to delay but it also affects the vibration mode of the emerging oscillations. Therefore, we conclude, that the *substructurability* of a system largely depends on the interface which, therefore, needs to be chosen carefully at the design stage of the experiment.

As for future research directions, an in-depth study of cable tension and other possible nonlinear effects and their influence on the substructured system would be of interest. Another valuable development will be to perform the discussed substructuring experiment, so that the issues related to the physical realization of such a test can be studied in detail. In this regard, it would also be interesting to evaluate the role of real-time noise in the actuator control for the overall accuracy of the substructure test.

## Data accessibility

Data are openly available from DOI: http://dx.doi.org/10.5523/bris.1nvlufa3lqc7n1a3u7xi1w0o4p.
